# Supplementary material for: ARCTIC-3D: automatic retrieval and clustering of interfaces in complexes from 3D structural information
Source: Commun Biol. 2024 Jan 6;7:49. doi: 10.1038/s42003-023-05718-w (PMC10771469; doi:10.1038/s42003-023-05718-w)
Supplement: Supplementary file 2 — Supplementary material [file 42003_2023_5718_MOESM2_ESM.pdf]

Supplementary Material for "ARCTIC-3D: automatic retrieval and clustering of interfaces in complexes from 3D structural information"

Marco Giulini[1], Rodrigo V. Honorato[1], Jesús L. Rivera[1], Alexandre M.J.J. Bonvin[1]

[1]Bijvoet Centre for Biomolecular Research, Faculty of Science - Chemistry, Utrecht University, Padualaan 8, 3584 Utrecht, CH, The Netherlands

Contents

1. Software Performance Analysis p.2

2. Understanding and visualizing angles and distances between interfaces p.5

3. On the clustering cutoff threshold p.8

4. Retention of alternative interfaces: an example p.10

5. Applying arctic3d-resclust to CPORT predictions p.11

Supplementary References p.12

1 Software Performance Analysis

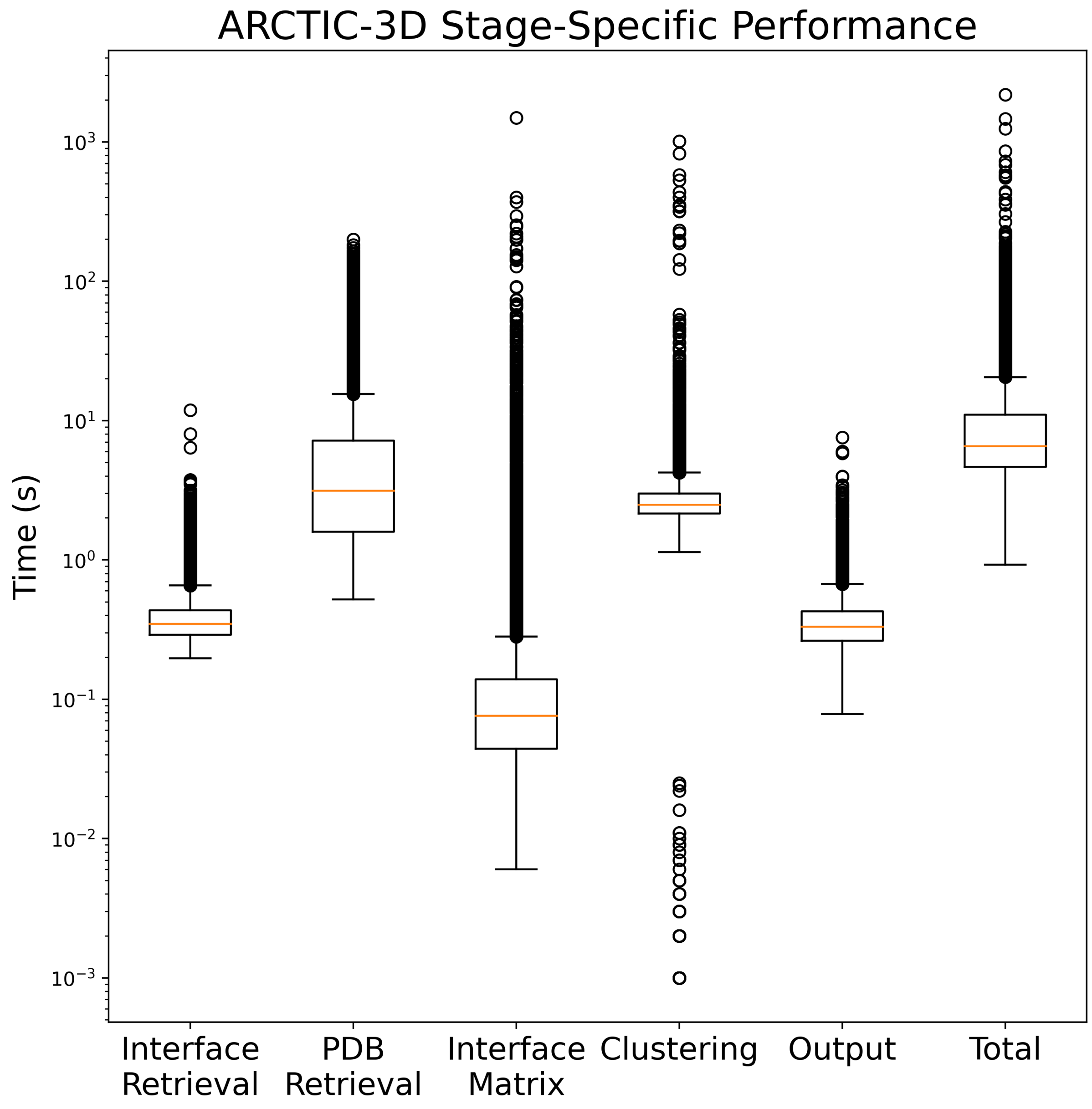

Supplementary Figure 1: Box plot showing the execution time (in logarithmic scale) proper to each step of the ARCTIC-3D protocol for the 23446 UNIPROT IDs for which interface information is available (see main text, Sec. UNIPROT-wide analysis). The *PDB retrieval* stage is usually the computational bottleneck of the procedure, accounting for more than 60 % of the execution time on average.

In this section we break down the performance of ARCTIC-3D based on the 23446 proteins discussed in the main text (Sec. UNIPROT-wide analysis). Those were the UNIPROT IDs for which interface information is available on the PDBe Graph Api.

Average and median execution times associated to the five different stages of the protocol, together with the overall execution time, are presented in Table 1, while Fig. 1 shows the corresponding boxplot.

Stage	Mean (s)	Median (s)
Interface Retrieval	0.4	0.35
PDB Retrieval	6.98	3.13
Interface Matrix	0.53	0.08
Clustering	2.81	2.48
Output	0.37	0.33
Total	11.09	6.52

Supplementary Table 1: Mean and median execution times for the five stages of the ARCTIC-3D protocol, together with the average and median overall execution time, calculated over the 23446 ARCTIC-3D runs on the UNIPROT swissprot database (see main text).

The *Interface Retrieval* step, in which interfaces are retrieved and parsed is never a problem in terms of computational time. The *PDB retrieval* stage, instead, is typically the bottleneck of the protocol, as the code has to retrieve and download several PDB files to pick the one retaining the highest amount of interfaces. The timing related to these steps are only indicative as they heavily depend on the network connection speed.

Execution speed of *Interface Matrix* and *Clustering* steps is linked to the number of retrieved interfaces, as these steps scale quadratically ($O(N^2)$ for *Interface Matrix*) or worse ($O(N^2 logN)$ for *Clustering*) with this number. Fig. 2 shows the scatter plot between *Interface Matrix* execution time and number of overall residues and interfaces. There is a dependency on the number of amino acids, given by the presence of more gaussian couplings in the dissimilarity calculation, but the main limiting factor is clearly given by the number of interfaces.

The *Output* stage takes typically a few hundreds milliseconds to complete, reaching the seconds timescale only when multiple, big PDB files have to be written to the disk.

The `pdb-to-use` and `chain-to-use` parameters allow to select the PDB file and chain to be retrieved in the *PDB retrieval* stage. If these values are defined, this stage's computational cost decreases dramatically, thus approximately halving ARCTIC-3D average execution time.

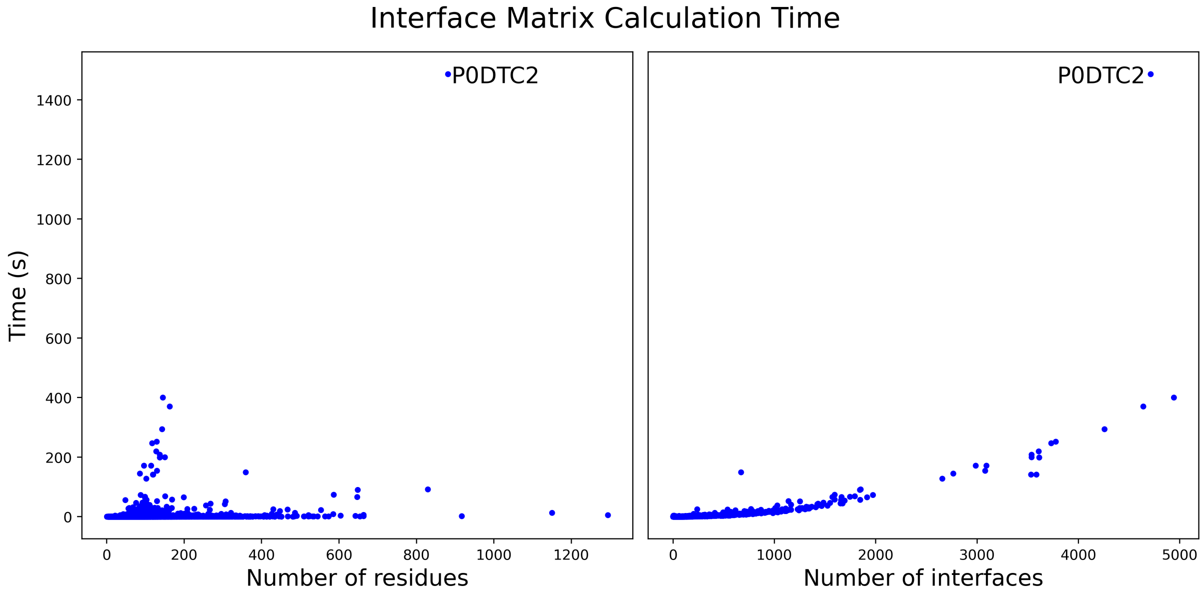

Supplementary Figure 2: Scatter plot between the execution time of the *PDB retrieval* step of the ARCTIC-3D workflow and the number of residues involved in the retrieved interfaces (left) and the number of retrieved interfaces (right). Although the execution speed of this step shows a slight dependency on the number of residues, the number of retrieved interfaces is the major factor responsible for longer execution times. The outlier of this graph (P0DTC2) is the *SARS-CoV-2 spike glycoprotein*.

2 Understanding and visualizing angles and distances between interfaces

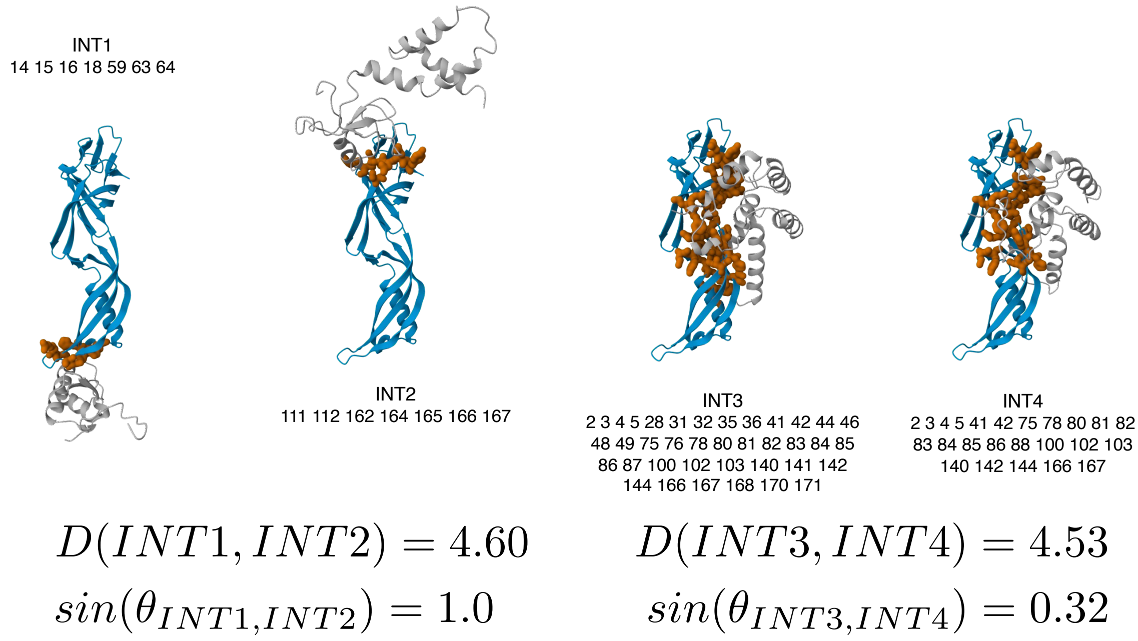

Supplementary Figure 3: Visualization of four different interfaces on the structure of the *DNA-directed RNA polymerase II RPB7 subunit* (UNIPROT ID P62487, light blue in the picture). Interface residues are highlighted in orange in each figure, while the corresponding interacting partner is represented in gray. INT1 and INT2 are small but biologically different interfaces located on two different sides of the protein. INT3 and INT4 are bigger interfaces that mediate the interaction of SUMO-1 with the *DNA-directed RNA polymerase II RPB4 subunit* (UNIPROT ID O15514). Although INT3 and INT4 do not share the totality of their amino acids, their dissimilarity should be low, while it should be higher for INT1 and INT2. The distance does not reflect this, giving two very similar values. The sine of the angle ($sin(\theta)$) is more effective, as it assigns the highest possible dissimilarity ($sin(\theta) = 1.0$) to the first pair, while identifying INT3 and INT4 as closely related ($sin(\theta) = 0.32$). Image produced with Molstar (1).

An important ingredient of the ARCTIC-3D protocol revolves around the definition of the quantity that should be used to discriminate between different interfaces. In the main text (Methods section) we proposed to use the sine of the angle between two interfaces rather than the distance between them. This is because this quantity is not robust with respect to differences in the numbers of amino acids between the various interfaces.

Fig. 3 visualizes this issue for four interfaces formed by UNIPROT ID P62487 (*DNA-directed RNA polymerase II subunit RPB7*). INT1 refers to the interface formed with UNIPROT ID P51948 (*CDK-activating kinase assembly factor MAT1*) in PDB file 6o9l (chain 3). INT2 is related to UNIPROT ID P61218 (*DNA-directed RNA polymerases I, II, and III subunit RPABC2*) and is retrieved from PDB 5iyc, chain F. Clearly these two interfaces are located in two different regions of the protein and, therefore, should be structurally separated.

INT3 and INT4 are two interfaces formed by UNIPROT ID P62487 with UNIPROT ID O15514 (*DNA-directed RNA polymerase II subunit RPB4*) in PDB files 6xre (chain D) and 6drd (chain D).

Although they differ by a few residues (INT3 has a few more amino acids in the lower domain), they share a very substantial fraction of interface residues. Biologically, they represent the same interface.

If we look at the numbers shown in Fig. 3 we can see how the sine of the angle properly describes the previous observations, while the distance does not. In fact, the distance between INT1 and INT2 would be approximately equal to the one between INT3 and INT4, thus contradicting the statement that the latter are extremely closer than the former. Note that the distance we are referring to is not the simple cartesian distance between the centers of the interfaces but is calculated according to Eq. 5 described in the Methods section of the main manuscript. The use of the angle solves this issue, as it only measures the overlap between interfaces, irrespectively of the number of amino acids forming them.

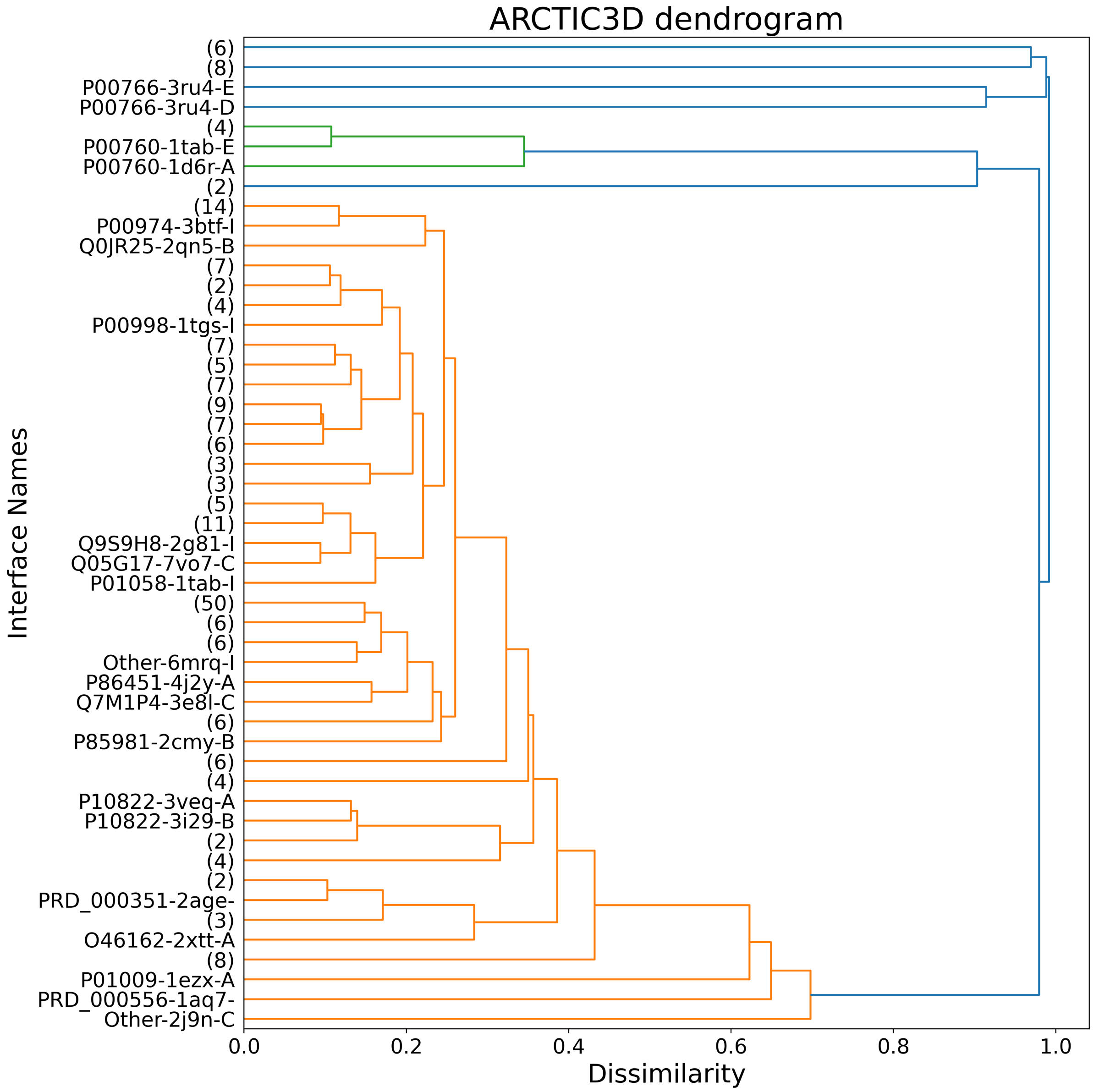

Supplementary Figure 4: Example dendrogram produced by ARCTIC-3D over the 228 retained interfaces of P00760. On the x-axis we can observe how the values of dissimilarity approach 1 as the inter-cluster distance approaches the maximum possibile dissimilarity. On the y-axis we can see some of the interface names (assigned with the `partner-pdb-chain` logic). Labels with round brackets (for example (6)) refer to group of interfaces that are already clustered together at that level of dissimilarity (typically very similar interfaces coming from the same PDB file).

3 On the clustering cutoff threshold

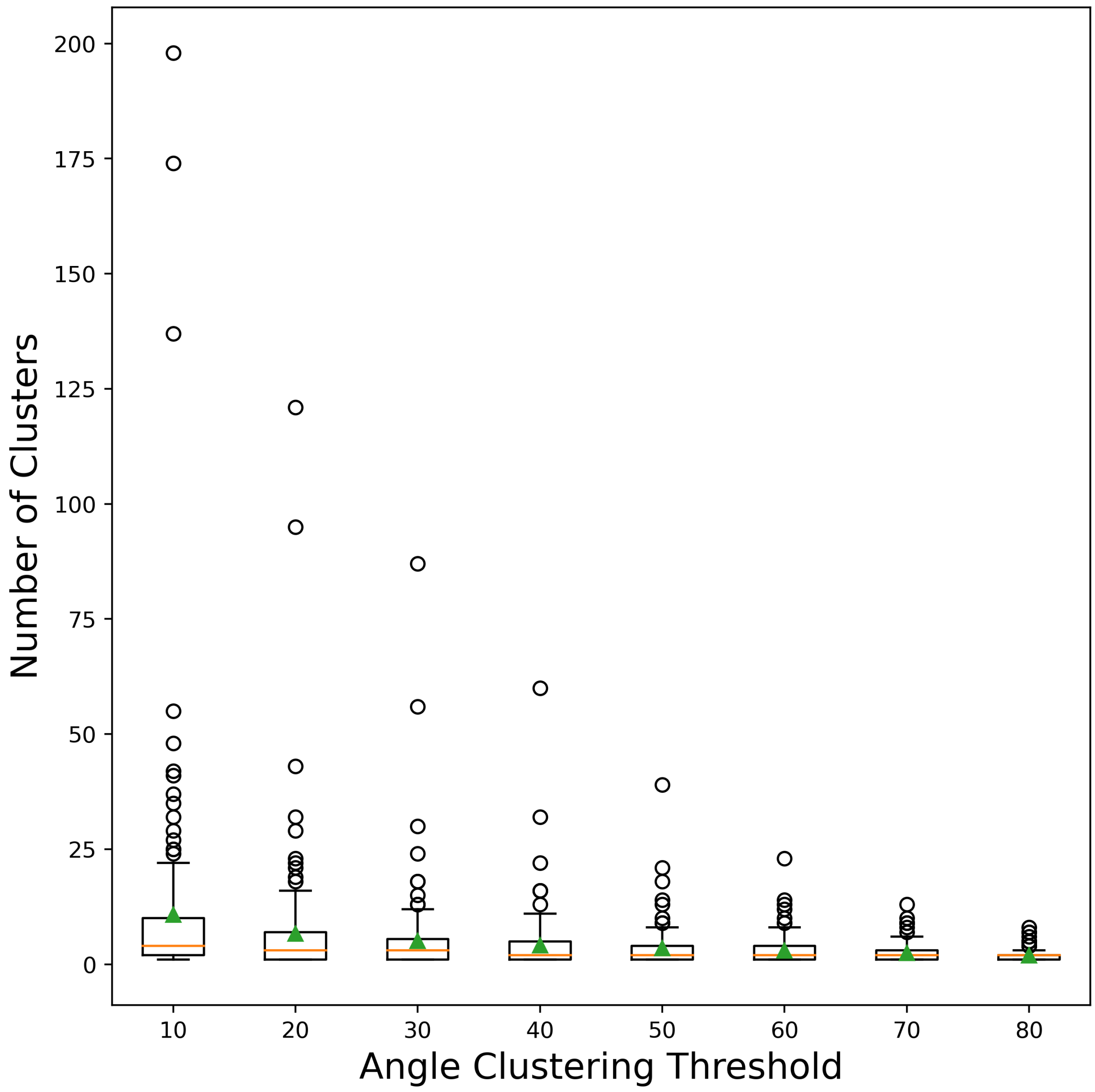

Supplementary Figure 5: Box plot showing the dependency of the number of clusters with the value of θ used in the clustering threshold. Orange lines represent median values, while green triangles represent average values.

The default value of the cutoff in ARCTIC-3D clustering is 0.866, namely the value of the sine of an angle θ equal to 60 degrees. Such threshold may not be perfect for all needs, as a stricter or a looser clustering may be more appropriate in some cases. Users can check the ARCTIC-3D output dendrogram (see Fig. 4 for an example) and adjust the `threshold` parameter to fine-tune the clustering.

Here we aim at illustrating the impact of the threshold on the generated clustering data for the proteins of the BM5 dataset (discussed in the main text, Sec. Results). We re-ran the analysis with eight, evenly spaced, values of the angle threshold, going from 10 to 80.

Fig. 5 shows the relationship between the angle clustering threshold and the number of generated clusters. When the threshold angle is lower than 50 the clustering procedure is very strict, thus giving rise to a high number of binding surfaces. In the limit case of $\theta = 10$, many proteins are associated to more than 25 clusters, thus making the cluster analysis cumbersome. Instead, high clustering thresholds ($\theta = 80$) tend to lump all the interfaces in very few binding surfaces. We find $\theta = 60$ to be a good compromise between these two scenarios.

From Fig. 5 it is also possible to understand how the same difference (e.g. 10 degrees) in the angle clustering threshold is typically more impactful at lower values, while being less significant at higher values (for example going from 70 to 80 degrees). Using the sine of such angle removes this dependency.

4 Retention of alternative interfaces: an example

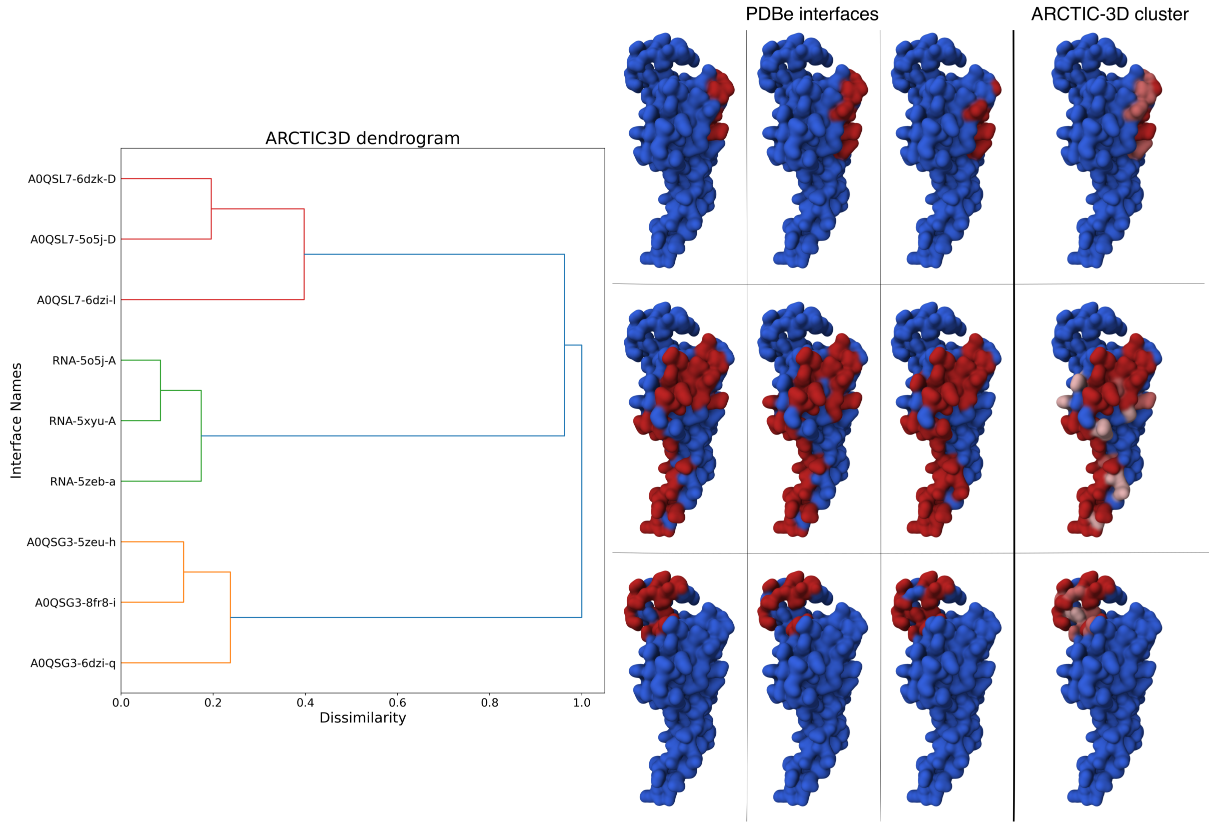

Supplementary Figure 6: Example application of ARCTIC-3D to nine interfaces retrieved for UNIPROT ID A0QSG6. The interfaces are compared to each other in the dendrogram (left) and divided in three binding surfaces that are localised in different regions of the protein (right). Image produced with Molstar (1).

Fig. 6 shows the results of the application of ARCTIC-3D on nine interfaces retrieved for the *Small ribosomal subunit protein uS5* (UNIPROT ID A0QSG6).

On the left side of the figure we see the dendrogram that illustrates how these interfaces are compared to each other and merged in clusters. On the right, the three interfaces composing each cluster are projected onto the reference structure (PDB 5xyu, chain E). The corresponding ARCTIC-3D clusters are shown, using the color coding described in the main text.

5 Applying arctic3d-resclust to CPORT predictions

As an example scenario for the `arctic3d-resclust` CLI, we retrieve a set of possibly interacting residues for pdb file 3HMR (2) using CPORT (3) with default settings. CPORT is a software dedicated to the prediction of protein-protein interface amino acids by combining up to six different predictors. Upon providing `arctic3d-resclust` with the structure and the list of possibly interacting amino acids, we obtain two clusters (see Table 2 and Fig. 7) containing two spatially separated sets of residues.

Group	Residues
Full set	39,40,41,42,44,45,57,58,59,60,61,62,76,77,78,79,<br>80,82,83,84,86,98,101,105,123,124,125,126,127
Cluster 1	39,40,41,42,44,45,105,123,124,125,126,127
Cluster 2	57,58,59,60,61,62,76,77,78,79,80,82,83,84,86,98,101

Supplementary Table 2: Example application of `arctic3d-resclust` to the PDB structure 3HMR. The set of residues provided by CPORT is divided in two groups using average linkage hierarchical clustering (4) with a threshold of 15 Å.

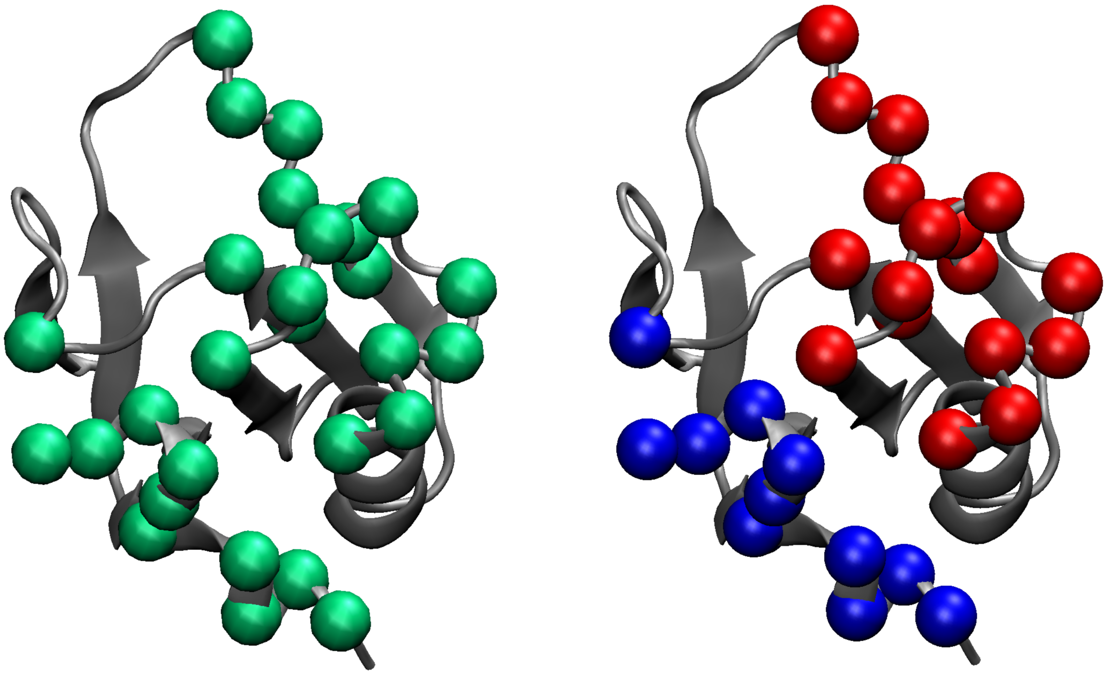

Supplementary Figure 7: Pictorial representation of the residue-based clustering performed by `arctic3d-resclust`. The amino acids labelled as likely interacting by CPORT are subdivided in two clearly separated patches on the protein surface. Image produced with VMD (5).

Supplementary References

[1] D. Sehnal, S. Bittrich, M. Deshpande, R. Svobodová, K. Berka, V. Bazgier, S. Velankar, S. K. Burley, J. Koča, and A. S. Rose, "Mol* viewer: modern web app for 3d visualization and analysis of large biomolecular structures," *Nucleic Acids Research*, vol. 49, no. W1, pp. W431–W437, 2021.

[2] W. D. Tolbert, J. Daugherty-Holtrop, E. Gherardi, G. Vande Woude, and H. E. Xu, "Structural basis for agonism and antagonism of hepatocyte growth factor," *Proceedings of the National Academy of Sciences*, vol. 107, no. 30, pp. 13264–13269, 2010.

[3] S. J. de Vries and A. M. Bonvin, "Cport: a consensus interface predictor and its performance in prediction-driven docking with haddock," *PloS one*, vol. 6, no. 3, p. e17695, 2011.

[4] R. R. Sokal, "A statistical method for evaluating systematic relationships.," *Univ. Kansas, Sci. Bull.*, vol. 38, pp. 1409–1438, 1958.

[5] W. Humphrey, A. Dalke, and K. Schulten, "Vmd: visual molecular dynamics," *Journal of molecular graphics*, vol. 14, no. 1, pp. 33–38, 1996.
